# Water Extract of *Piper longum* Linn Ameliorates Ovariectomy-Induced Bone Loss by Inhibiting Osteoclast Differentiation

**DOI:** 10.3390/nu14173667

**Published:** 2022-09-05

**Authors:** Dong Ryun Gu, Hyun Yang, Seong Cheol Kim, Youn-Hwan Hwang, Hyunil Ha

**Affiliations:** 1 KM Convergence Research Division, Korea Institute of Oriental Medicine, Yuseong-daero 1672, Yuseong-gu, Daejeon 34054, Korea; 2 Korean Convergence Medicine Major KIOM, University of Science & Technology (UST), 1672 Yuseongdae-ro, Yuseong-gu, Daejeon 34054, Korea

**Keywords:** *Piper longum* Linn, osteoporosis, ovariectomy, osteoclast

## Abstract

*Piper longum* linn has traditionally been used for the treatment of respiratory and gastrointestinal disorders in India. Although various pharmacological effects of *P. longum* have been studied, its effects on bone have not been clearly elucidated. Therefore, this study examined the inhibitory effect of the water extract of *P. longum* Linn (WEPL) on osteoclast differentiation. WEPL directly affected the osteoclast precursors and suppressed osteoclast differentiation in vitro. In addition, the expression levels of c-Fos and nuclear factor of activated T cells 1, a critical transcription factor for osteoclastogenesis, were significantly downregulated by WEPL via the suppression of the receptor activator of nuclear factor (NF)-κB ligand-induced mitogen-activated protein kinase and NF-κB signaling pathways. Consistent with the in vitro results, oral administration of WEPL (100 and 300 mpk) to ovariectomized mice for six weeks relieved the OVX-induced bone loss. We also identified phytochemicals in WEPL that are reported to exert inhibitory effects on osteoclastogenesis and/or bone loss. Collectively, the findings of our study indicate that WEPL has an anti-osteoporotic effect on OVX-induced bone loss by diminishing osteoclast differentiation, suggesting that it may be useful to treat several bone diseases caused by excessive bone resorption.

## 1. Introduction

Bones play an important role in supporting the whole body, protecting the internal organs, generating blood cells, storing minerals, and sustaining bone remodeling to enable proper functioning [1]. In normal physiological bone remodeling, a balance between bone-resorbing osteoclasts and bone-forming osteoblasts is pivotal [1], and osteocytes, which are other bone cells embedded in the bone matrix, orchestrate the formation and functions of these two cells [2,3]. Any deviation in this balance can lead to bone diseases, such as osteoporosis (OP), osteopetrosis, and Paget disease [1,4,5].

Among bone cells related to bone remodeling, osteoclasts are multinucleated giant cells that are able to resorb bone. Osteoclasts begin to differentiate from monocyte/macrophage lineage precursor cells when receptor activator of the nuclear factor-κB ligand (RANKL), a principal cytokine for osteoclastogenesis, binds to its receptor RANK on osteoclast precursors [6,7]. Thereafter, the recruitment of adaptor molecules of RANK, such as tumor necrosis factor receptor-associated factor 6 (TRAF6), induces mitogen-activated protein kinases (MAPK) and NF-κB signaling, leading to increased expression of essential transcription factors, c-Fos and nuclear factor of activated T cells 1 (NFATc1), for osteoclast differentiation [8,9,10].

Natural products have been receiving increasing attention for the treatment of OP [11,12]. Compared to bone formation, OP caused by undue osteoclastic bone resorption is characterized by deteriorated bone mass and flimsy micro-architecture of the bone tissue, which leads to severe bone fracture [13]. Through our preliminary screening of extracts of medicinal plants used in Korean medicine for their anti-osteoporotic activity, we found that the water extract of the unripe fruit of *Piper longum* Linn (WEPL) inhibited osteoclast differentiation.

*P. longum* grows indigenously in South and Southeast Asia and is cultivated in the tropical and subtropical regions of Asia and the Pacific Islands [14]. Unripe fruit of *P. longum* has been broadly used for respiratory disorders, gastrointestinal disorders, aphrodisiac, emmenagogues, circulatory stimulants, and analgesics in Ayurvedic medicine [14,15]. Although previous studies have shown that *P. longum* possesses numerous biological activities, including anticancer [16], antioxidant [17], anti-inflammatory [18], antimicrobial [19], and antidiabetic [20] effects, the pharmacological properties of *P. longum* on bone metabolism are still unclear. In this regard, determining the new biological activity of *P. longum*, this study was focused on investigating a biological effect of *P*. *longum* extract against OP. For the purpose of this study, we examined the inhibitory effects of the WEPL on osteoclast differentiation and the protective effects of WEPL on bone loss using the ovariectomized mouse model.

## 2. Materials and Methods

### 2.1. Materials

Recombinant RANKL and macrophage colony-stimulating factor (M-CSF) were supplied as previously described [21,22]. Acetonitrile, water, formic acid, bicinchoninic acid assay (BCA) kit was obtained from Thermo Fisher Scientific (Waltham, MA, USA). Antibodies against phospho-p38 (T180/Y182), p38, phospho-extracellular signal-regulated kinase (p-ERK, T202/Y204), ERK, phospho-IκBα (S32), IκBα, phospho-Jun N-terminal kinase (p-JNK, T183/Y185), JNK, and β-actin were purchased from Cell Signaling Technology (Danvers, MA, USA). Anti-c-Fos, anti-NFATc1, and secondary antibodies were obtained from Santa Cruz Biotechnology (Dallas, TX, USA). RNeasy kit was obtained from Qiagen (Hilden, Germany). High-capacity cDNA reverse transcription kit and universal PCR master mix were purchased from Applied Biosystems (Foster City, CA, USA).

### 2.2. Preparation of WEPL

WEPL was supplied by the National Development Institute of Korean Medicine (Gyeongsan, Korea) and stored in the herbarium (voucher number #JW60) of the KM Convergence Research of the Korea Institute of Oriental Medicine. Briefly, air-dried unripe fruits of *P. longum* (500 mg) were extracted using distilled water (3.5 L) under reflux for 3 h. The extracts were filtered and lyophilized. The powder was dissolved in distilled water and filtered through a 0.2 μm filter prior to use.

### 2.3. Preparation of Bone Marrow-Derived Macrophages (BMMs) and Cell Viability Assay

Bone marrow cells (BMCs) were prepared from tibiae and femora of 8-week-old C57BL/6J mice. The cells were incubated in α-minimal essential medium (α-MEM; Hyclone, Logan, USA) containing 10% heat-inactivated fetal bovine serum (FBS; Gibco) with M-CSF (20 ng/mL) for one day. Non-adherent BMCs were collected and incubated on untreated cell culture dishes with M-CSF (60 ng/mL) for three days. Adherent cells were considered as BMMs. BMMs were cultured with WEPL for 24 h, and the cytotoxicity of WEPL was analyzed by Cell Counting Kit-8 (Dojindo Molecular Technologies, Rockville, MD, USA).

### 2.4. In Vitro Osteoclastogenesis

For in vitro osteoclast differentiation, a co-culture system of MLO-Y4 cells, an osteocyte-like cell line, and BMMs, osteoclast precursor cells, was used. Briefly, MLO-Y4 cells (1 × 10^3^ cells/well) and BMMs (4 × 10^4^ cells/well) in a 96-well plate were co-cultured in α-MEM containing 10% FBS with 1α,25-dihydroxyvitaminD_3_ (VitD_3_, 10 nM) for 5 days. The culture medium was replaced with fresh medium containing VitD_3_ on day 3. To directly induce the differentiation of osteoclast precursor cells, BMMs (1 × 10^4^ cells/well) were cultured with RANKL (50 ng/mL) and M-CSF (60 ng/mL) for four days, and the culture medium was exchanged on day three. To assess the total tartrate-resistant acid phosphatase (TRAP) activity, cells were fixed with 10% formalin for 20 min and then permeabilized with 0.1% Triton X-100 in DPBS for 30 min. Thereafter, the cells were treated with 150 μL of TRAP buffer (0.12 M sodium acetate and 50 mM sodium tartrate) containing 1 mg/mL of 4-nitrophenyl phosphate disodium (Sigma-Aldrich, St. Louis, MO, USA) at 37 °C for 15 min. Afterwards, the 100 μL of each solution was transferred into new plates containing 100 μL of 0.1 N sodium hydrate to terminate the reaction. The absorbance was read at 405 nm wavelength using SPECTRAmax^TM^ 340 (Molecular Devices Corporation, San Jose, CA, USA). For TRAP-staining, 0.1 mg/mL of naphthol AS-MX phosphate and 0.5 mg/mL of fast red violet LB salt (Sigma-Aldrich) were dissolved in TRAP buffer. Cells were incubated with staining solution for 30 min at room temperature. After TRAP staining, cells were washed briefly and photographed.

### 2.5. Western Blotting

The cells were lysed in PRO-PREP^TM^ (iNtRON biotechnology, Sung-nam, Korea). Protein quantification was performed using a BCA kit. Lysates (30 μg) were denatured by heating at 100 °C for 5 min. Thereafter, the lysates were separated by sodium dodecyl sulfate-polyacrylamide gel electrophoresis and transferred onto polyvinylidene fluoride membranes (Bio-Rad Laboratories, Hercules, CA, USA) using a western blot apparatus. The membranes were blocked for 2 h with 5% skim milk in TBS-T (50 mM Tris, 150 mM NaCl pH 7.6, and 0.1% Tween-20) and then incubated overnight at 4 °C with a 1:1000 dilution of the specific antibody. The 1:5000 dilution of Horseradish peroxidase-conjugated IgG was used as the secondary antibody. The target proteins were visualized with a ChemiDoc Touch imaging system and Image Lab software V5.2.1 (Bio-Rad Laboratories).

### 2.6. Quantitative Real-Time Polymerase Chain Reaction (PCR)

The RNeasy Mini kit was employed to prepare total RNA according to the manufacturer’s instructions. Thereafter, cDNA was synthesized from 2 μg of total RNA using a high-capacity cDNA reverse transcription kit. TaqMan gene expression assays for c-Fos (Fos, Mm00487425_m1), NFATc1 (Mm00479445_m1), dendritic cell-specific transmembrane proteins (Dcstamp, Mm01168058_m1), ATPase H^+^ transporting V0 subunit d2 (Atp6v0d2, Mm00656638_m1), cathepsin K (Ctsk, Mm00484036_m1), interferon regulatory factor-8 (IRF-8, Mm00492567_m1), v-maf avian musculoaponeurotic fibrosarcoma oncogene homolog B (MafB, Mm00627481_s1), RANKL (Tnfsf11, Mm00441908_m1), osteoprotegerin (OPG, Tnfrsf11b, Mm00435454_m1), and 18S rRNA (18s, Hs99999901_s1) were used for PCR reaction. PCR amplification was performed using TaqMan Universal Master Mix II and the Quant Studio 6 Flex real-time PCR system (Applied Biosystems). All results were normalized to 18S rRNA as an internal reaction control. Relative quantification was performed using the ∆∆Ct method.

### 2.7. Ultrahigh-Performance Liquid Chromatography–Tandem Mass Spectrometry (UHPLC–MS/MS) Analysis

The characterization chemical ingredients of *P. longum* Linn were carried out as described previously study [23,24]. Dionex UltiMate 3000 system (LabX, Midland, ON, Canada) with a Thermo Q-Exactive mass spectrometer (UHPLC-MS/MS, Thermo Fisher Scientific) were used for analysis. All tested compounds were separated using an Acquity BEH C18 column (100 × 2.1 mm, 1.7 μm) with water and acetonitrile containing 0.1% formic acid. The mass spectra of *P. longum* Linn analyzed using a heated electrospray ionization source. The full scan mass spectra were acquired in positive ion mode at a scan range of 100–1500 *m*/*z* in data-dependent MS2 scan mode. Data acquisition and analysis were based on Xcalibur and TraceFinder software (Thermo Fisher Scientific). The reference standards, magnoflorine and piperine, were purchased from Targetmol (Wellesley Hills, MA, USA).

### 2.8. Animal Study

Six-week-old female C57BL/6J mice (Japan SLC Inc., Shizuoka, Japan) were accommodated in a specific pathogen-free environment in which the facility is under controlled temperature (22 ± 2 °C), humidity (55 ± 5%), and regular photoperiod (12 h light/dark cycle). The mice had free access to drinking water and a standard chow diet during one week of acclimatization to ovariectomized (OVX) or sham-operated conditions. All mice were classified into four groups (*n* = 6): sham, OVX, OVX mice administered WEPL 100 mg/kg (WEPL-L), and OVX mice administered WEPL 300 mg/kg (WEPL-H). During the six weeks, WEPL was administered by oral gavage once daily, and the standard chow was changed to a normal rodent diet containing 10 kcal% fat (D12450B, Research Diets, New Brunswick, NJ, USA). After 7 h of fasting, the mice were sacrificed to prepare samples.

### 2.9. Micro-Computed Tomography (Micro-CT) Analysis

The microarchitecture of the distal femur in the mice was scanned using micro-CT (SkySacn 1276, Bruker, Kontich, Belgium). SkyScan NRecon (version 1.7.42, Bruker) was used to reconstruct the images after scanning. The region of the femur commencing at a distance of 80 µm from the growth plate and extending across 150 cross-sections (0.8 μm section thickness) in the proximal direction was selected as the volume of interest using SkyScan CTAn (version 1.20.3.0, Bruker). Bone morphometric parameters including trabecular bone volume fraction (BV/TV, %), trabecular bone mineral density (BMD; g/cm^3^), trabecular number (Tb.N, mm), trabecular thickness (Tb.Th, mm), and trabecular separation (Tb.Sp, mm) were assessed.

### 2.10. Statistical Analysis

Statistical analyses were performed using GraphPad Prism, version 8 (GraphPad, CA, USA). Results are presented as the mean ± standard deviation for in vitro study or as mean ± standard error of the mean for the in vivo study. Between-group differences were calculated using one-way analysis of variance (ANOVA) followed by Dunnett’s post hoc test or two-way ANOVA followed by Sidak’s post hoc test to correct for multiple comparisons.

## 3. Results

### 3.1. WEPL Restrains Osteoclastogenesis in BMMs and Osteocyte-like Cell Co-Culture

**Figure 1:**
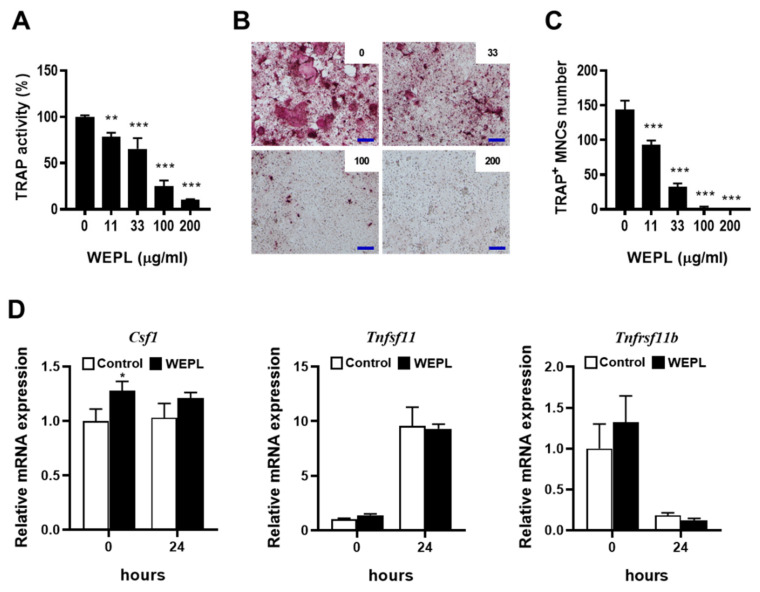
Effects of WEPL on osteoclast differentiation in BMM-MLO-Y4 co-culture. BMMs and MLO-Y4 cells were co-cultured with the indicated concentrations of WEPL in the presence of VitD_3_ (10 nM) for five days. (**A**) Total TRAP activity. (**B**) Microscope images of TRAP-stained cells. scale bar, 100 μm (**C**) TRAP-positive multinucleated cells (TRAP^+^ MNCs) containing more than three nuclei were counted as osteoclasts. (**D**) MLO-Y4 cells were cultured with WEPL (100 μg/mL) or distilled water as a vehicle for 3 h and then treated with VitD_3_ (10 nM) for 24 h. The mRNA expression of *Csf1, Tnfsf11* and *Tnfrsf11b* was examined via real-time PCR. * *p* < 0.05, ** *p* < 0.01, *** *p* < 0.001 vs. control.

When co-cultured with osteoclast precursors, osteocyte-like cells have been shown to support osteoclast differentiation by providing RANKL, and VitD_3_ can further promote the ability of MLO-Y4 cells [25]. We examined the effect of WEPL on osteoclast differentiation in a co-culture system of osteoclast precursors BMMs and MLO-Y4 cells. Consistent with previous reports [25], treatment with VitD_3_ induced osteoclastogenesis in the co-culture, which was suppressed by WEPL in a dose-dependent manner (Figure 1A–C). To investigate the mechanism by which WEPL inhibits osteoclast differentiation, we examined whether WEPL affects the expression of RANKL and the decoy receptor OPG in MLO-Y4 cells. VitD_3_ markedly increased the mRNA expression of RANKL (encoded by *Tnfsf11*) and decreased that of OPG (encoded by *Tnfrsf11b*). Treatment with WEPL at 100 μg/mL, which greatly reduced osteoclast differentiation, did not affect VitD_3_-stimulated expression of RANKL and OPG (Figure 1D). These results suggest that WEPL does not affect the capacity of MLO-Y4 to support osteoclast differentiation.

### 3.2. WEPL Suppresses the Differentiation of Osteoclast Precursors

**Figure 2:**
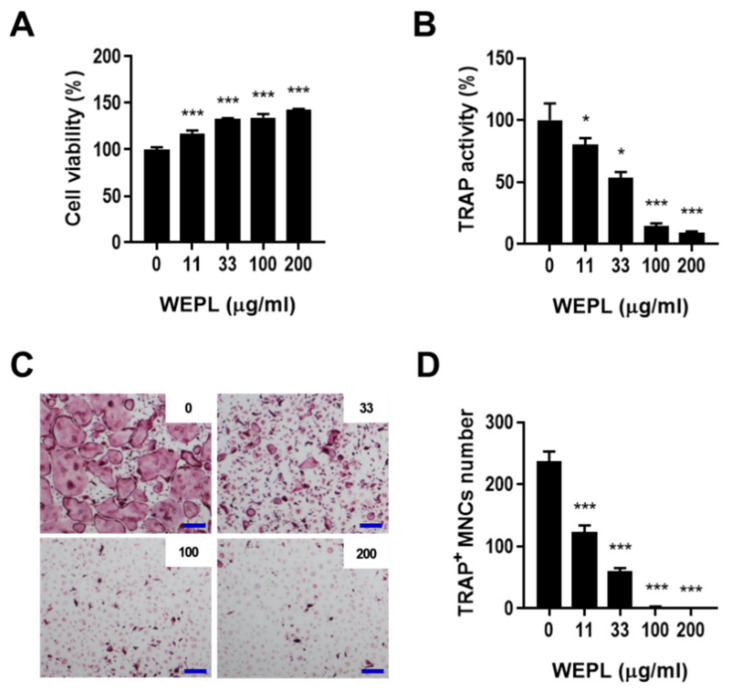
Effect of WEPL on the differentiation of osteoclast precursors. (**A**) BMMs were incubated with WEPL (0–200 μg/mL) for one day, and cell viability was analyzed. (**B**–**D**) BMMs were cultured with or without WEPL in the presence of RANKL (50 ng/mL) and M-CSF (60 ng/mL). (**B**) Total TRAP activity. (**C**) Representative microscope images of TRAP-stained osteoclasts. Scale bar, 100 μm. (**D**) The number of TRAP^+^ MNCs. * *p* < 0.05, *** *p* < 0.001 vs. control non-treated with WEPL.

To verify that WEPL inhibits osteoclast differentiation by directly influencing its precursors, BMMs were cultured with or without WEPL in the presence of M-CSF and RANKL, which can fully mature osteoclast precursors [6,26]. WEPL did not exert any cytotoxicity on BMMs but upregulated the viability of BMMs (Figure 2A). Similarly to the results obtained in the co-culture system, total TRAP activity and the number of TRAP^+^ MNCs were decreased by WEPL in a dose-dependent manner (Figure 2B–D). These results indicate that WEPL diminishes osteoclast differentiation by directly acting on osteoclast precursors rather than on supporting cells.

### 3.3. WEPL Attenuates the Expression of Osteoclastogenic Transcription Factors

**Figure 3:**
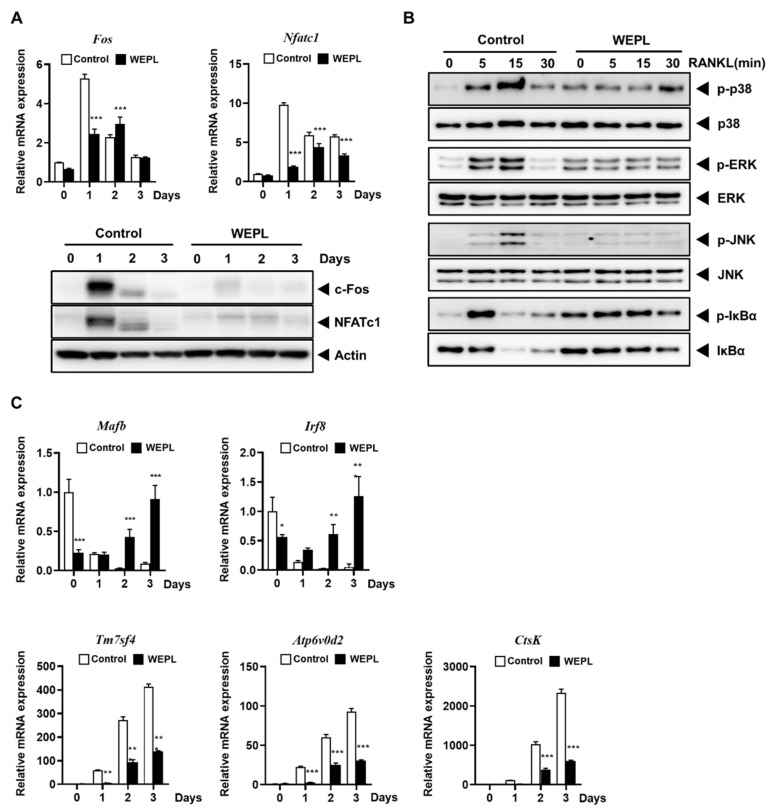
Effects of WEPL on the expression levels of osteoclastogenic transcription factors and osteoclast-specific genes. BMMs were incubated with M-CSF and RANKL in the presence or absence of WEPL (100 μg/mL) for the indicated times. (**A**) The mRNA and protein levels of c-Fos and NFATc1 were analyzed by PCR and western blotting. (**B**) BMMs were stimulated with RANKL, 3 h after pre-treatment with WEPL. The expression levels of MAPKs (p38, ERK, and JNK) and IκBα was detected using western blot. (**C**) The mRNA expression of negative regulators (*Mafb* and *Irf8*) and osteoclast-specific genes (*Tm7sf4*, *Atp6v0d2*, and *CtsK*). * *p* < 0.05, ** *p* < 0.01, *** *p* < 0.001 vs vehicle.

To investigate the mechanism underlying the inhibitory effect of WEPL on the differentiation of osteoclast precursors, we analyzed the expression of c-Fos and NFATc1, which are critical transcription factors required for osteoclast differentiation [10,27]. Compared with the control group, mRNA and protein expression of c-Fos was dramatically decreased on day 1, while mRNA and protein expression of NFATc1 was downregulated on days 1 and 2 in the WEPL-treated group (Figure 3A). The binding of RANKL to its receptor RANK triggers recruitment of TRAF6 and stimulates various signal transduction pathways, such as the MAPKs and NF-κB pathways, followed by the induction of NFATc1 and c-Fos expression [8,9]. Therefore, we investigated the early signal transduction pathways induced by RANKL. As shown Figure 3B, pretreatment with WEPL suppressed not only MAPKs activation (p38, ERK, and JNK), but also IκBα degradation, even though it stimulated basal IκBα phosphorylation. Given the roles of MAPKs and NF-κB signaling pathways in c-Fos and NFATc1 expression and osteoclastogenesis, our results illustrate that WEPL inhibits c-Fos and NFATc1 expression by impeding RANKL-induced activation of MAPKs and NF-κB pathways.

In addition, we confirmed mRNA levels of genes reported as osteoclast negative regulators to suppress NFATc1 expression, *Mafb* and *Irf8* [28,29]. The relative mRNA levels of both genes were obviously increased by WEPL-treatment (Figure 3C). As NFATc1 transcribes osteoclast-specific genes to boost osteoclast differentiation and function, we investigated the expression of genes related to osteoclast fusion, *Tm7sf4* and *Atp6v0d2* [30,31], and osteoclast function, *CtsK* [32]. Consistent with the decrease in NFATc1 expression, WEPL significantly downregulated mRNA expression of *Tm7sf4*, *Atp6v0d2*, and *CtsK* (Figure 3C). Collectively, these results indicate that WEPL attenuates c-Fos and NFATc1 expression and the expression of osteoclast-specific genes, leading to the inhibition of osteoclast differentiation.

### 3.4. WEPL Ameliorates OVX-Induced Bone Loss in Mice

**Figure 4:**
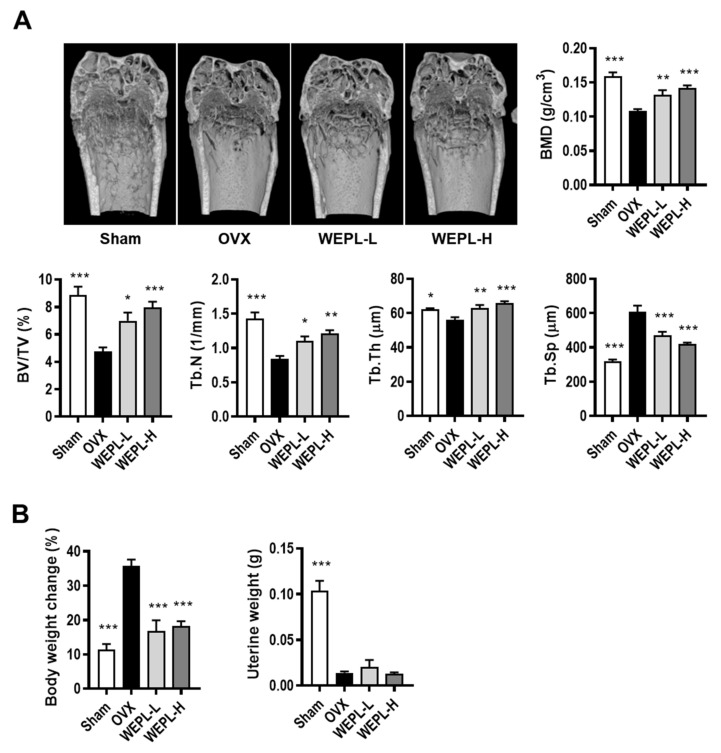
Effects of WEPL on OVX-induced bone loss. After ovariectomy, WEPL was administered at two concentrations, 100 mg/kg/day (WEPL-L) and 300 mg/kg/day (WEPL-H) for 6 weeks. (**A**) Representative images of distal femur scanned by micro-CT and morphometric analysis of trabecular bone (BMD, BV/TV, Tb.N, Tb.Th, Tb.Sp) (**B**) Measurement of body weight change and uterine weight. * *p* < 0.05, ** *p* < 0.01, *** *p* < 0.001 vs. OVX.

Furthermore, we examined whether WEPL protects against osteoclast-mediated pathological bone loss in OVX mice, a typical model of postmenopausal OP [33]. As shown in Figure 4A, micro-CT analysis demonstrated that ovariectomy caused a significant decrease in BMD, BV/TV, Tb.Th, and Tb.N, and a remarkable increase in Tb.Sp, indicating a significant impairment of trabecular bone in the femur. In contrast, OVX mice treated with low or high concentrations of WEPL displayed considerable amelioration of bone loss, reversing the OVX-induced changes in bone parameters. As shown in Figure 4B, WEPL administration also reduced OVX-induced body weight gain by a similar level as SHAM but did not affect uterine weight. These results demonstrate that these ameliorative effects of WEPL on OVX-related bone loss and weight change are independent of its possible phytoestrogenic action.

### 3.5. Phytochemical Constituents of WEPL

**Figure 5:**
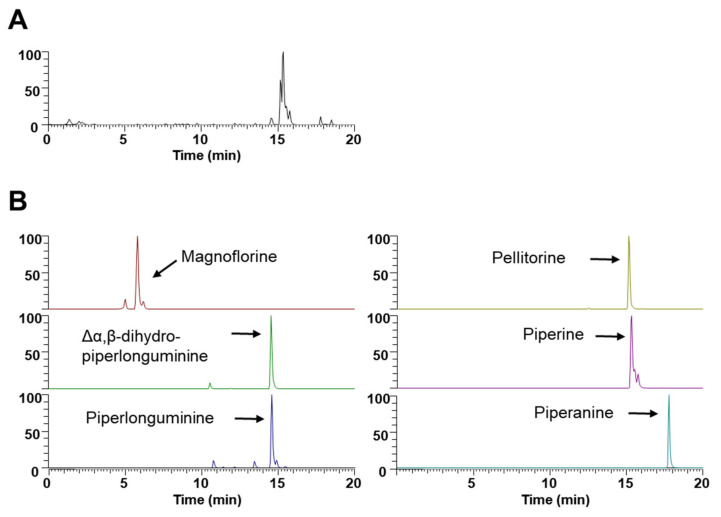
UHPLC–MS/MS analysis of WEPL. (**A**) Base peak chromatogram; (**B**) Extracted ion chromatogram of identified phytochemicals.

To investigate which components contribute to the inhibitory mechanisms of WEPL on osteoclastogenesis and OP, we carried out UHPLC–DAD–MS/MS to obtain phytochemical profile of WEPL. As detected via mass spectra and retention times, WEPL was composed of six alkaloids: magnoflorine, Δα,β-dihydro-piperlonguminine, piperlonguminine, pellitorine, piperine, and piperanine (Figure 5 and Table 1). Magnoflorine and piperine were identified by comparison with authentic standards [34]. The other four alkaloids detected in this study were consistent with those identified in previous reports [24,35]. Previous studies have shown that magnoflorine relieves inflammatory osteolysis in vivo and RANKL-induced osteoclastogenesis in vitro via downregulation of MAPKs and NF-κB signaling [36], and piperine also exerts recovery effects on OVX-related bone loss and improves osteogenic differentiation in MC3T3-E1 cells [37,38]. In addition, pellitorine has also been reported to act as an antagonist of the ion channel TRPV1 [39], connected with osteoclast differentiation and function [40,41,42], implying that pellitorine might have an inhibitory effect on osteoclastogenesis. Collectively, these results suggest that the constituents of WEPL, namely magnoflorine, piperine, and pellitorine, may account for the inhibitory effects of WEPL on osteoclast differentiation and OVX-associated bone loss.

## 4. Discussion

Previous studies have demonstrated that osteocytes physiologically orchestrate osteoclastogenesis by the secretion of several cytokines such as M-CSF, RANKL, and OPG [43,44,45]. In this study, WEPL inhibited VitD_3_-stimulated osteoclast differentiation in a co-culture of osteoclast precursors and osteocyte-like MLO-Y4 cells, whereas it did not affect VitD_3_-stimulated expression of RANKL and OPG in MLO-Y4 cells, suggesting its direct inhibitory action on osteoclast precursors. In fact, treatment of osteoclast precursors with WEPL inhibited osteoclast differentiation by suppressing RANKL-induced up-regulation of positive regulators such as c-Fos and NFATc1 and down-regulation of negative regulators, MafB and Irf8.

The most typical animal model of estrogen-deficient OP is produced in mice by OVX. These mice with surgically removed bilateral ovaries gradually lose cancellous bone and uterine weight [33]. It therefore is suitable for evaluating anti-osteoporotic effect of candidate agents. In particular, the internal and external structure of bone can be observed and analyzed in great detail using micro-CT, thereby providing improved the diagnosis and evaluation of OP [45]. In the present study, micro-CT analysis revealed that administration of WEPL reduced estrogen-deficient bone loss without estrogenic effects. Thus, these data suggest that WEPL can be a therapeutic candidate for estrogen-deficient OP.

Easily available, inexpensive and helpful *P. longum* is used as flavor in cooking and is traditionally used medicine [46]. Because of its widespread use, it is considered safe for moderate consumption. The safety of the *P. longum* extract has been demonstrated by various studies [46,47]. Acute oral toxicity studies on the ethanolic extract of *P. longum* L. fruit were conducted in mice. The mice administered the extract of 3 g/kg/day showed no significant morphological, spermatogenic, and hematological changes [47]. In addition to acute studies, no adverse effects were reported in research conducted with extracts of *P. longum* at various doses (25–1000mg/kg/day) and durations (1–6 weeks) [48,49,50,51]. In the present study, no mortality or no abnormal symptoms were found in mice administered with WEPL (100 and 300 mg/kg/day). However, since plant extracts have large differences in composition depending on the extraction method, further studies on toxicity and side effects are needed.

Various pharmacological activities of *P. longum* including cancers, ischemic heart diseases, and diabetes are considered to be attributed mainly to its alkaloid components [15,16,17,18,19,20]. In our study, six alkaloids including magnoflorine and piperine having bone protective effects were identified as the main components of WEPL. The estimation of ADMET (Ab-sorption, Distribution, Metabolism, Excretion, Toxicity) is critical at the early stage of drug development as most candidates fail at clinical trials due to in-adequate ADMET properties. In a previous study on the pharmacokinetic properties of *P. longum*, in silico estimation showed that the percentage of human intestinal absorption of 136 phytochemicals found in *P. longum* is more than 90% [14]. The alkaloids, piperine, piperlonguminine, Δα,β-dihydro-piperlonguminine, pellitorine, and piperanine found in WEPL are included in the 136 phytochemicals. In addition, the five alkaloids also satisfy the Lipinski’s rule of five for drug-likeness evaluation [14]. Taken together, these results suggest that WEPL and its alkaloid components are useful for anti-osteoporotic drug development.

## 5. Conclusions

In this study, we found that WEPL did not affect the capability of osteocyte-like MLO-Y4 cells to support osteoclastogenesis, whereas it impeded osteoclastogenesis by suppressing RANKL-induced intracellular signaling, particularly the MAPKs and NF-κB pathways, and subsequently suppressed the expression of NFATc1 and c-Fos in osteoclast precursor cells. In an animal model of menopausal OP, WEPL alleviated bone loss and weight change, without phytoestrogenic action. We also identified the phytochemicals, magnoflorine and piperin, in WEPL, which had inhibitory effects on osteoclast formation and bone loss. Altogether, our findings suggest that WEPL is a promising herbal candidate for the prevention and treatment of bone diseases caused by excessive bone resorption.

## Figures and Tables

**Table 1:** Identification of the phytochemicals in WEPL by UHPLC-ESI-MS/MS analysis.

No	Retention Time (min)	[M]^+^/[M + H]^+^ (*m/z*)		Elemental Composition	Error (ppm)	MS/MS Fragments (*m*/*z*)	Identification
		Estimated	Calculated				
1	5.8	342.1700	342.1698	C_20_H_24_NO_4_	−0.4374	342.17, 297.112, 265.086	Magnoflorine *
2	14.54	276.1594	276.1591	C_16_H_21_NO_3_	−1.0914	203.07, 161.06, 135.044	Δα,β-dihydropiperlonguminine
3	14.59	274.1438	274.1436	C_16_H_19_NO_3_	−0.6741	201.055, 135.044	Piperlonguminine
4	15.15	288.1594	288.1592	C_17_H_21_NO_3_	−0.8342	203.07, 135.044	Pellitorine
5	15.34	286.1438	286.1436	C_17_H_19_NO_3_	−0.7525	201.055, 135.044	Piperine *
6	17.79	224.2009	224.2007	C_14_H_25_NO	−0.7208	224.201, 168.138, 151.112	Piperanine

*, compared with the retention time and MS spectral data of authentic standards.

## Data Availability

All relevant data are within the paper.
